# End of life in patients attended by pediatric palliative care teams: what factors influence the place of death and compliance with family preferences?

**DOI:** 10.1007/s00431-023-04870-z

**Published:** 2023-03-09

**Authors:** Maria José Peláez-Cantero, Jose Miguel Morales-Asencio, Álvaro Navarro-Mingorance, Aurora Madrid-Rodriguez, Ángela Tavera-Tolmo, Olga Escobosa-Sánchez, Ricardo Martino-Alba

**Affiliations:** 1 Department of Paediatric Palliative Medicine, Maternal-Child of Malaga, Regional University Hospital. University of Málaga, Málaga, Spain; 2 University of Málaga. Malaga Biomedical Research Institute (IBIMA), Málaga, Spain; 3 Department of Paediatric Palliative Medicine, Niño Jesus University Children’s Hospital, Madrid, Spain; 4 Department of Paediatric Palliative Medicine, Son Espases University Hospital, Mallorca, Spain; 5 Department of Paediatric Palliative Medicine, Virgen de Las Nieves University Hospital, Granada, Spain

**Keywords:** Advance directives, Child mortality, Decision-making, Infant death palliative care, Right to die, Terminal care

## Abstract

Each year, more than 8 million children worldwide require specialized palliative care, yet there is little evidence available in pediatrics on the characteristics of the end of life in this context. Our aim is to analyze the characteristics of patients who die in the care of specific pediatric palliative care teams. This is ambispective, analytical observational, multicenter study conducted between 1 January and 31 December 2019. Fourteen specific pediatric palliative care teams participated. There are 164 patients, most of them suffering from oncologic, neurologic, and neuromuscular processes. The follow-up time was 2.4 months. The parents voiced preferences in respect of the place of death for 125 of the patients (76.2%). The place of death for 95 patients (57.9%) was at the hospital and 67 (40.9%) was at home. The existence of a palliative care team for over 5 years is more likely to be related to families voicing preferences and their fulfillment. Longer follow-up times by pediatric palliative care teams were observed in families with whom preferences regarding the place of death were discussed and in patients who died at home. Patients who did not receive home visits, when the pediatric palliative care team did not provide full care and when preferences regarding the place of death were not discussed with parents, were more likely to die in the hospital.

*  Conclusions*: Advance planning of end-of-life care is one of the most important aspects of pediatric palliative care. The provision of services by the teams and the follow-up time are related to parents’ expressed preferences and the place of death.

**Table:** 

**What is Known:** *• Various studies have shown how the availability of pediatric palliative care services improves the quality of life of patients and their families while reducing costs.* *• The place of death is an important factor influencing the quality of end-of-life care for dying people. The increase in palliative care teams increases the number of deaths in the home and having this care available 24/7 increases the probability of dying at home.*	
**What is New:** *• Our study identifies how a longer follow-up time of patients by palliative care teams is significantly associated with death at home and with express and comply with the preferences expressed by families.* *• Home visits by the palliative care team increase the likelihood that the patient will die at her home and that the preferences expressed by the palliative care team families will be cared for.*

**What is Known:**

*• Various studies have shown how the availability of pediatric palliative care services improves the quality of life of patients and their families while reducing costs.*

*• The place of death is an important factor influencing the quality of end-of-life care for dying people. The increase in palliative care teams increases the number of deaths in the home and having this care available 24/7 increases the probability of dying at home.*

**What is New:**

*• Our study identifies how a longer follow-up time of patients by palliative care teams is significantly associated with death at home and with express and comply with the preferences expressed by families.*

*• Home visits by the palliative care team increase the likelihood that the patient will die at her home and that the preferences expressed by the palliative care team families will be cared for.*

## Introduction

The number of children in need of pediatric palliative care (PPC) is increasing [1]. Each year, more than 21 million children worldwide require a palliative approach, and of these, more than 8 million require specialized palliative care [2].

Specialized pediatric palliative care (SPPC) is an integrative model of care for children with life-threatening illnesses that aims to ease suffering, improve child and family quality of life, and support families in delineating their goals of care and making decisions accordingly [3]. Palliative care is an essential component of universal health coverage; several studies have shown how the availability of PPC services improves the quality of life of patients and their families [4, 5] and also reduces costs [6–8].

Worldwide, only 5.7% of countries provide well-developed pediatric palliative care [9] and few WHO European Region countries consistently report a high level of integration in PPC [10]. In 2014, the Spanish Ministry of Health published the document “Pediatric Palliative Care in the National Health System: Care Criteria” [11] with the aim of improving the quality of care provided to advanced and terminally ill patients and their families.

Although there has been an increase in the number of resources for palliative care in recent years, there is little evidence in pediatrics about the population served, the models of care, the organizational characteristics of these resources, the services provided and their effectiveness, nor of the level of orientation towards shared decision-making with patients and families [12].

The aim of this study is to analyze the characteristics of patients who die in the care of specific pediatric palliative care teams (PPCT) in Spain, and to determine what factors influence the place of death and compliance with family preferences.

## Methods

Ambispective, analytical observational, multicenter study, conducted between 1 January and 31 December 2019. Fourteen PPCTs from across the country participated in the data collection (Table 1).

**Table 1:** Organizational characteristics of pediatric palliative care teams, mortality rates, and number of patients included

**Hospital (year of creation of the PPCT)**	Type of care*	**Option of home visits**	**Number of patients cared for by the PPCT in 2019**	**Number of deceased patients cared for by the PPCT in 2019** ***n***	**Mortality rate 2019 (%)**	**Number of patients included in the study** ***n* (%)**
San Juan de Dios Hospital, Barcelona (1992)	Full	Yes	119	59	49.5%	42 (25.6%)
Infantil Niño Jesús University Hospital Madrid (2008)	Full	Yes	127	40	31.4%	40 (24.4%)
Málaga Regional University Hospital (1999)	Partial	Yes**	59	17	28.8%	17 (10.4%)
Virgen del Rocío University Hospital Seville (2016)	Partial	Yes	63	16	25.3%	16 (9.8%)
Son Espases University Hospital Mallorca (2013)	Partial	Yes	79	9	11.3%	9 (5.5%)
Virgen de las Nieves University Hospital Granada (2018)	Partial	Yes**	50	8	16%	8 (4.9%)
Cruces University Hospital Bilbao (2012)	Partial	Yes	52	6	11.5%	6 (3.7%)
Miguel Servet University Hospital Zaragoza (2017)	Partial	Yes	87	6	6.8%	6 (3.7%)
Virgen de la Arrixaca University Hospital Murcia (2009)	Partial	Yes	35	5	14.2%	5 (3.0%)
Torrecárdenas University Hospital Almería (2014)	Partial	Yes	34	5	14.7%	5 (3.0%)
Parc Taulí University Hospital Sabadell (2016)	Partial	Yes	50	3	6%	3 (1.8%)
Toledo Virgen de la Salud Complex (2015)	Partial	No	58	7	12%	3 (1.8%)
General University Hospital Alicante (2008)	Partial	Yes	96	3	3.1%	3 (1.8%)
Nuestra Señora de Candelaria University Hospital Tenerife (2018)	Partial	No	35	1	2.8%	1 (0.6%)

^*^Full: care 24 h/7 days a week. Partial: remaining care

^**^Home care coverage is not provided to all patients in the province

### Study participants

All patients who received care from the PPCTs during the study period were included and were followed up until death under the responsibility of the team’s care, at which time data collection was performed. We had a clinical contact point (pediatrician or nurse) in each PPCT for data collection which was carried out using a standardized form and the analysis of medical records. Deceased patients for whom no data were obtained were identified as missing.

Most of the PPCT in our country work in a homogeneous way. They provide patient care regardless of where the patient is, hospital or home.

Patient and family variables were collected such as sex, age, date of birth, baseline disease, date of diagnosis, date of inclusion under the PPCT, symptoms 7 days prior to death, drugs and devices in the last week and 24 h prior to death, date of death, presence of sedation and reason, cause of death, place of death, and family preferences for place of death. Variables related to the provision of services by the teams were also collected, such as year of creation, type of care (full: 24 h/7 days a week or partial: all others), and the option of making home visits.

The “Pediatric Complex Chronic Disease Classification System,” version 2, developed by Feudtner [13], was used to describe the underlying disease.

### Statistical analyses

We performed the analysis by using Statistical Package for Social Sciences 26 and Jamovi 2.3. An exploratory analysis was performed to determine the nature of the distributions and to obtain statistics on central tendency, dispersion, and frequencies. The normality of the distributions was tested using the Kolmogorov–Smirnov test and asymmetry and kurtosis analysis.

Bivariate statistics were performed using the chi-square test with Fisher correction when appropriate for qualitative variables. For the analyses of medical device utilization as a function of the existence of an expression of intent, crude and adjusted odds ratios were calculated using the Cochran-Mantel–Haenszel method. Mean differences were also performed using the Mann–Whitney *U* test and Spearman correlations.

Finally, multivariate logistic regression models were constructed to predict the place of death as well as compliance with advance directives. A process of intentional selection of predictors was carried out for those variables that had shown a link in the bivariate analysis, as well as those that clinically had plausibility and in the Wald test had a *p* value < 0.25 [14]. Covariates were eliminated from the model if they did not contribute significance or act as confounders. At the end of the iteration process, discarded variables were re-evaluated in the final model to ascertain if they made a significant contribution to the presence of other variables.

## Results

During the study period, the PPCTs cared for 944 patients, of whom 185 died, with a median specific mortality rate of 13.1% (IQR 19.6). After excluding patients for whom no data were obtained, the final sample consisted of 164 patients (sample loss of 11.4%), 84 females (51.2%) and 80 males (48.8%). The median age at diagnosis was 1.4 years (IQR 7.4), the median age at the inclusion of patients under the PPCT was 5.2 years (IQR 10.9), the median time from disease progression to death was 9.6 months (IQR 38.4), and the median follow-up time of the patient and family by the PPCT was 2.4 months (IQR 10.8), with no differences between the groups based on the diagnosis. The median age at death was 6.7 years (IQR 10.8).

The underlying diseases are shown in Table 2, with most of the cases deriving from oncological, neurological, and neuromuscular processes. Besides, we assigned groups according to the Association for Children with Life-threatening or Terminal Conditions and Their Families/Royal College of Pediatrics and Child Health (ACT/RCPCH, hereinafter referred to as ‘‘ACT’’) [15] (Table 3).

**Table 2:** Baseline disease

**Baseline disease**	***n* (%)**
Malignancy	79 (48.2%)
Neurological and neuromuscular	39 (23.8%)
Metabolic	15 (9.1%)
Prematurity and neonatal	14 (8.5%)
Congenital or genetic defects	12 (7.3%)
Others	5 (3.1%)

**Table 3:** Association for children with terminal conditions group

**ACT group**	***n* (%)**
ACT 1: Feasible curative treatment but possibility of failure	52 (31.7)
ACT 2: Possible life prolonging treatment allowing normal activities	3 (1.8)
ACT 3: Progression without curative treatment options	69 (42.1)
ACT 4: Irreversible non-progressive conditions with susceptibility to life-shortening complications	40 (24.4)

In total, 12 PPCTs (85.7%) had the option to make home visits, although in some cases, not all patients were visited due to geographical dispersion, and only 2 had the option of full care: 24 h/7 days a week (Table 1).

**Fig. 1:**
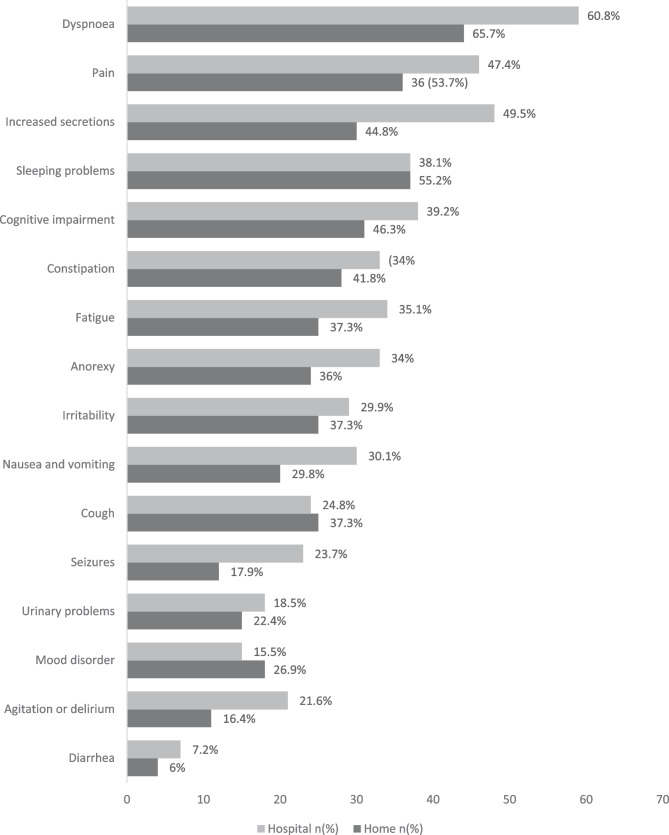
Total frequency of symptoms at the end of life and by place of death

In the last week of life, the symptoms that appeared most frequently were dyspnoea, pain, increased secretions, sleep problems, and cognitive impairment (Fig. 1).

One hundred forty-one patients (86%) were aided by a device, most commonly oxygen therapy (101 patients, 61.6%) and intravenous (66 patients, 40.2%). The subcutaneous route was used in 29 patients (17.7%), rising to 55 (33.5%) in the last 24 h.

The reason for death was a progression of the underlying disease in 101 patients (61.6%), comorbidity in 51 (31.1%), and the cause of death unexpected in only 12 (7.3%).

Sedation was applied in 31.7% of patients in the last week and rose to 50.6% in the last 24 h, the most frequent reasons of sedation being death agony in 40 patients (24.4%) and treatment of refractory symptoms in 34 patients (20.7%).

The hospital was the place of death of 95 patients (57.9%) while 67 (40.9%) died at home; 2 patients (1.2%) died during the transfer from their home to the hospital. Mortality rates were significantly different among hospitals (Kruskal-Wallis test *χ*^2^: 159; ε^2^: 0.97; *p* < 0.001) like oncological underlying disease (*χ*^2^: 22.7; Cramer’s V: 0.37; *p* = 0.045). Consequently, an analysis of the mortality rates in the presence of oncological underlying disease was carried out and no significant results were found.

Parents expressed preferences as to the place of death for 125 patients (76.2%); of these, 70 families (56%) preferred death to take place at home. Preferences were complied with in 119 cases (99.2% of those who had expressed them; *χ*^2^ = 129.9; *p* < 0.01), with a significant positive association between expressing a preference and having it complied with (Cramer’s v = 0.9, *p* < 0.01).

In the bivariate analysis, it was detected that families who received a home visit or who were being followed by a PPCT that had existed for more than 5 years tended to prefer for their child to die at home (*χ*^2^ = 24.7, *p* < 0.01 and *χ*^2^ = 11.4; *p* = 0.003, respectively). PPCT experience of more than 5 years is also related to is more likely to be related to families expressing preferences and that to those preferences being complied with (*χ*^2^ = 8.7; *p* = 0.003 and *χ*^2^ = 7.4, *p* < 0.007).

Longer PPCT follow-up times were observed for patients who died at home (median 0.5, IQR: 1.3 vs. median: 0.2; IQR: 0.7) (*z* =  −2.24; *p* = 0.025), and longer follow-up times were also found in the case of families with whom the place of death was discussed (median 0.3, IQR: 1.1 vs. 0.08, IQR: 0.58) (*z* = 3.1; *p* = 0.006).

As for symptoms, no differences were observed in their distribution according to the place of death (Fig. 1).

We found a relationship between the type of care full/partial and the place of death. Thus, in respect of PPCTs providing full care, patients more often died at home than those with partial care (*χ*^2^ = 3,7; 59.7% vs. 40.3%, *p* = 0.039).

When analyzing the presence of sedation in the last 24 h in respect of the place of death, it was observed that patients who died in the hospital were sedated in 62.9% of cases (*n* = 65), compared to 33.3% (*n* = 22) of those who died at home (*χ*^2^ = 13.7; *p* < 0.01).

In the multivariate analysis, we found, that those patients who did not receive home visits were more likely to die in the hospital (OR 7.91 95% IC: 1.86 to 33.69), when preferences regarding the place of death had not been discussed with parents (OR 9.33 95% IC: 1.94 to 44.72), when the PPCT did not provide full care or their physicians had not made visits outside the hours of coverage (in case of partial care) (OR 2.75 95% IC: 1.01 to 7.45), if the patient no longer received food by mouth the previous week (OR: 7.04 95% IC: 2.63 to 18.89), and if the patient had no sedation the 24 h prior to death (OR: 4.34 95% IC: 1.74 to 10.63) (Table 4).

**Table 4:** Factors associated with place of death in the hospital

**Predictor**	***B***	***p***	**OR (95%CI)**
Constant	−2.95	< .001	0.05 (0.01 to 0.21)
Gender			
Male-Female	0.99	0.026	2.71 (1.13 to 6.53)
Age of death	0.02	0.617	1.02 (0.95 to 1.09)
Option of visits to the patient’s home:			
No - Yes	2.07	0.005	7.91 (1.86 to 33.69)
Extra service:			
Yes - No	−1.48	0.061	0.23 (0.05 to 1.07)
Expressed preferences about place of death:			
No - Yes	2.23	0.005	9.33 (1.94 to 44.72)
Full attention			
0–1	1.02	0.048	2.75 (1.01 to 7.45)
Sedation the day before death			
Yes - No	1.47	0.001	4.34 (1.74 to 10.63)
Oral feeding 1 week before death			
No - Yes	1.95	< .001	7.04 (2.63 to 18.89)

McFadden’s *R*^2^: 0.39, VIF range: 1.09 to 1.33, Tolerance range: 0.75 to 0.94

In a second model, we found that family preferences for place of death are more likely to be met in those patients with longer follow-up time by the PPCT (OR 1.76 IC 95%: 1.03 to 3.02), if patient home visits are made (OR 6.59 IC 95%: 2.77 to 15.65), and when the cause of death is comorbidity (OR 20.13 IC 95%: 3.44 to 117.62) or progression of the baseline disease (OR 39.79 IC 95%: 6.80 to 232.62) (Table 5). We observed that 95.5% (*n* 64) of patients whose preferences were met died at home compared to 56.7% (*n* 55) who died in the hospital, which is statistically significant (*p* < 0.005, OR 0.61).

**Table 5:** Factors associated with complying with family preferences

	*B*	*p*	OR (95%CI)
Gender (female)	−2.90	0.477	0.74 (0.32 to 1.68)
Age at death	−0.12	0.706	0.99 (0.92 to 1.05)
PPCT follow-up time	0.56	0.040	1.76 (1.03 to 3.02)
Receiving visits at home	1.89	< 0.001	6.59 (2.77 to 15.65)
Cause of death			
Comorbidity	3.00	0.001	20.13 (3.44 to 117.62)
Baseline disease progression	3.68	< 0.001	39.79 (6.80 to 232.62)

## Discussion

The death of a child is a devastating and tragic event for all those involved: the healthcare providers who are called on to address the child’s complex care needs, the family members distraught by grief, but above all the children who pay the highest price [16].

Palliative care for patients who require it is a right recognized by international organizations [17–19] that should be guaranteed to all children and their families in order to avoid as much futile or excessively burdensome practices as therapeutic abandonment.

### Main findings and implications

Recent prevalence studies demonstrate how the majority of children and adolescents living with a life-limiting condition (LLC) have congenital anomalies and neurological disorders [19], with cancer being the underlying disease in only 20% of children seen [20] by PPCTs. However, in our study, the majority of patients who died during follow-up by a PPCT were suffering an oncological disease as their baseline disease. This may be chiefly due to two reasons: on the one hand, cancer patients have the shortest survival time after being transferred to a PPCT [21], so it is possible that these teams care for many patients with these characteristics in end-of-life PPC, as has been seen in other publications [22], even though their follow-up time is short. On the other hand, there is probably an institutional bias towards the provision of services to oncology patients as opposed to other types of processes, which is supported by previous research reporting that existing palliative care provision is mainly directed to children with cancer [23]. Thus, parents of children with non-oncological diseases report greater unmet needs than parents of children with oncological diseases [24], which continues to highlight the differences in support that these two groups receive which are better established for cancer. Therefore, one of the key objectives in end-of-life strategies is to extend palliative care to patients with non-cancer diagnoses.

One important aspect of the palliative care approach is the planning of care, which has been shown to be beneficial to the child and family [25]. Pediatric advance care planning seeks to ensure end-of-life care conforming to the patients/their families’ preferences [26], including the location in which patients and their families wish to receive care towards the end of life. It is important to discuss the possible expected evolution throughout the course of the disease. End-of-life care should begin to be addressed early enough for the family to have time to prepare [27, 28] and become aware of death as one of the options, especially if the disease has a long trajectory. The end of life and its environment should be prepared and defined according to the child’s wishes and available resources.

Our follow-up time of the patient and family by the PPCT was slightly more than 2 months, less than previously described in other studies [29], without finding differences in terms of underlying disease. One of the most common barriers described in the literature [30] is that the responsible physicians think that the family is not ready to receive PPC and therefore delay or prevent this referral. There are studies [31] that describe how parents, with follow-up time by the PPCT, become increasingly skilled and adept at supporting care, managing symptoms, and administering medications to their child. Our study identifies how longer care by the PPCT is significantly associated with death at home and with the family expressing its preferences and those preferences being complied with. For this reason, early referral of patients to PPCTs is important in order to get to know the child and family and thus explore future prospects, anticipate more gradually, and incorporate family preferences regarding their child’s care. Key aspects for this early referral to PPCTs suggested by some authors focus on the formation of a formal PPC program, the launch of an educational campaign, and a PPCT expansion plan [32].

However, after creating PPCTs, they must also be maintained over time. The professionals who make up the PPCTs feel that “no formal ratification of the intervention by management” and “unsettled organisation” are limitations [33]. Our data highlight something not previously described in the literature, specifically that the more well-established PPCTs have a bearing on both the place of care and on talking to the family about their preferences.

Place of care and place of death are important factors influencing the quality of end-of-life care of dying people and often the latter as quality indicators in the evaluation of pediatric palliative care. Although international organizations state that family-centered home care is the goal of pediatric palliative care [23], the majority of terminally ill children die in hospitals [34]. As described in the literature, the proportion of deaths at home varies widely among populations with frequencies ranging from 7% in South Korea [34] to 45% in England [35] or 86% in Germany [36]. Our sample is situated in the middle. However, we should be cautious as epidemiological data on the place of death are not necessarily representative of the preferred location of death.

The publication of a recent meta-analysis reveals how the older age of the child and cancer diagnoses appear to be independent predictors of home death among children [37]. This relationship with the underlying disease had also been found in other studies [38]. However, in our sample, we found no significant relationship between age or baseline disease and place of death. The latter can be explained by the fact that in Spain most of the PPCT services have been based on initiatives outside oncology, and although we cannot ignore the fact that for patients with diseases other than cancer, there are barriers to inclusion in PPCT, such as the less predictable nature of most non-malignant diseases and the associated difficulty in identifying a terminal stage [39], once overcome, clinicians feel able to assume the end of life at home in these patients as well.

In addition, it has been shown that increased PPCT increases the number of deaths at home [40] and that without PPC provision, there was a long-term trend of dying away from home [41]. Families should have a choice of where end-of-life (EOL) care is provided with home as one option. Having a PPCT offering 24 h/7 days care [21, 42] increases the probability of dying at home, a relationship also found in our study and with an even greater association if professionals who provided out-of-hours care are included, although previously published studies describe working outside working hours as “exhausting” and not sustainable in the long term [43].

Regarding family decision-making about the place of care at the end of life and death, these are affected by personal, interpersonal, temporal, and disease-related factors [44]. In agreement with previous studies, those families who had made a decision showed a preference for death to occur at home [45, 46]: 56% in our study. But not only do we have to pay attention to what the parents want, but also to how the parents make a decision when given a choice about their child’s death and place of death. Parents should be provided with timely, detailed, and immediate information about their child’s diagnosis, clinical status, and prognosis in an appropriate and safe environment and should be invited into the decision-making process as each party will need the other to achieve their common goal: to give the sick child the best possible option [47].

### Strengths

The cohort included patients managed in the period under study from 14 different geographical areas, which guarantees good external validity.

### Limitations

The heterogeneity in mortality rates between centers. Also, data collection was based on the review of health records, with the limitations that this method entails [48].

## Conclusion

In summary, policies should be developed to allow people to die in their preferred place, ensuring that high-quality care is available wherever they are [38]. In addition, the chance to plan the place of death may be a better indicator of high-quality, more inclusive end-of-life care that is better aligned with palliative care principles [49].
